# Is Social Categorization Spatially Organized in a “Mental Line”? Empirical Evidences for Spatial Bias in Intergroup Differentiation

**DOI:** 10.3389/fpsyg.2018.00152

**Published:** 2018-02-15

**Authors:** Fabio Presaghi, Marika Rullo

**Affiliations:** Dipartimento di Psicologia dei Processi di Sviluppo e Socializzazione, Sapienza Università di Roma, Rome, Italy

**Keywords:** social categorization, spatial biases, spatial agency bias, SNARC effect, ingroup–outgroup

## Abstract

Social categorization is the differentiation between the self and others and between one’s own group and other groups and it is such a natural and spontaneous process that often we are not aware of it. The way in which the brain organizes social categorization remains an unresolved issue. We present three experiments investigating the hypothesis that social categories are mentally ordered from left to right on an ingroup–outgroup continuum when membership is salient. To substantiate our hypothesis, we consider empirical evidence from two areas of psychology: research on differences in processing of ingroups and outgroups and research on the effects of spatial biases on processing of quantitative information (e.g., time; numbers) which appears to be arranged from left to right on a small–large continuum, an effect known as the spatial-numerical association of response codes (SNARC). In Experiments 1 and 2 we tested the hypothesis that when membership of a social category is activated, people implicitly locate ingroup categories to the left of a mental line whereas outgroup categories are located on the far right of the same mental line. This spatial organization persists even when stimuli are presented on one of the two sides of the screen and their (explicit) position is spatially incompatible with the implicit mental spatial organization of social categories (Experiment 3). Overall the results indicate that ingroups and outgroups are processed differently. The results are discussed with respect to social categorization theory, spatial agency bias, i.e., the effect observed in Western cultures whereby the agent of an action is mentally represented on the left and the recipient on the right, and the SNARC effect.

“Us and Them and after all we’re only ordinary men…”

Pink Floyd, “Us and Them” lyrics (1973)

## Introduction

Social categorization involves the classification of oneself and others, often unconsciously or without intention, as members of social groups on the basis of shared attributes such as ethnicity, physical features, or even psychological traits (Stangor et al., 1992; Kunda and Spencer, 2003; Abrams, 2012). Self Categorization Theory (SCT; Turner et al., 1987, 1994) posits that people categorize themselves by exaggerating or emphasizing the perceived similarity amongst members of their group (reduction of differences) and, correspondingly, by emphasizing the differences between their group and members of the outgroup (meta-contrast principle, Turner et al., 1987; Oakes et al., 1999). Hence when an “ordinary man” become “us,” the intergroup differentiation prompts a contrast with “them.” People’s tendency to synchronize with their social environment on the base of salient social cues has important consequences for their working self-concept (Kawakami et al., 2012). Social identity theory (SIT; Tajfel and Turner, 1979) postulates that when people identify with a social group or when group membership is salient, they no longer perceive themselves as just ‘ordinary people’ but as interchangeable members of that group (Turner et al., 1987, 1994; Hogg and Tindale, 2008). Hence when categorized as a group member, people are generally motivated to strive to maintain a positive ingroup identity and to perceive their own group as morally superior to other groups (Tajfel and Turner, 1979; Oakes and Turner, 1980; Ellemers et al., 2008) or having more positive attributes and better social status (Sachdev and Bourhis, 1987, 1991; Spears and Manstead, 1989; Jost and Banaji, 1994). Furthermore, both SCT and SIT (Tajfel and Turner, 1979; Turner, 1982; Turner et al., 1987) suggest that the simple act of categorizing people as ingroup or outgroup is sufficient to generate ingroup favoritism and outgroup derogation (Doise and Sinclair, 1973; Brewer, 1979, 1999; Turner et al., 1979).

### Cognitive Processes Underlying the Representation of Self in Social Groups

Although there has been extensive research into how social categorization and social identification lead to ingroup favoritism at the expense of outgroups (Rubin and Hewstone, 1998; Hewstone et al., 2002), less is known about the cognitive processes underlying the representations of social groups. Is social categorization so pervasive that the processes follow a specific cognitive organization?

Some studies assessing reaction times (RTs) in an ingroup–outgroup categorization task (RT interference in self-description task, Aron et al., 1991; Smith et al., 1999) have found that not only do people perceive ingroup members as less ‘distant’ than outgroup members (Brewer, 1991; Smith et al., 1999), but the self is the “habitual reference point” for comparisons and similarity judgments (Srull and Gaelick, 1983; Smith et al., 1999, p. 874). Some authors have also investigated how social categorization alters estimates of physical distance (Xiao and Van Bavel, 2012) and have shown that people tend to overestimate the distance between a domestic (ingroup) and a foreign (outgroup) location shown on a map, relative to the distance between two domestic locations or two foreign locations (Burris and Branscombe, 2005). Schubert and Otten (2002) suggested that the degree of overlap between self and ingroup or outgroup can be represented by the metaphorical mapping of the self to the group onto the spatial dimension:

We enter or leave a group; we distance ourselves from a group or are in the inner circle. Finally, we can be simply in a group, which then becomes an ingroup: The interrelation constructs (Higgins and Chaires, 1980, p. 353) in and out denoting ingroup and outgroup are spatial metaphors.

So it seems reasonable to conclude that ingroups and outgroups are processed differently and that the self functions as an exemplary member of the ingroup in this process. Does this mean that when distinguishing between ingroup and outgroup by reference to oneself as an ingroup member we represent social categories in spatial terms?

### Spatial Biases and Their Relationship to Social Categories

The concept of spatial organization of social constructs is not new; examples include embodied cognition (Barsalou, 1999; Richardson et al., 2003; Smith, 2005) and spatial agency bias (SAB; Chatterjee, 2002; Maass et al., 2009). In particular, SAB is the notion that cultures with a left-to-right (LR) script are characterized by an LR scanning habit and by a standard ordering of subject (agent) and object in building (active) phrases. Both factors contribute to culture-specific spatial biases; for example, the perception that time flows LR (Gevers et al., 2004) or mental representation of numbers with smaller numbers on the left (Dehaene et al., 1993), but they also favor the representation of the actions related to intergroup situations in a way that reflects the default ordering of subject and object in a phrase, such that more agentic actors are mentally represented on the left and the less agentic actors on the right (Maass et al., 2014). Moreover, SAB has also been found to be closely related to stereotypic expectations: stereotypically more agentic actors tend to be represented toward the left (Maass et al., 2014). Maass et al. (2014) argued that these spatial biases often influence communication between social groups in a subtle way and we believe that another kind of social bias is involved when social category rather than agency is salient. In particular, we hypothesized that when social category becomes salient, the representation of social category is organized such that the category to which one belongs is located on the left, near the self.

### Hypotheses

One of the most stable and frequently replicated spatial biases is the spatial-numerical association of response codes (SNARC) effect first described by Dehaene et al. (1993) and Wood et al. (2008). The SNARC effect is that in a parity-judgment RT task, reactions to large target numbers are faster when the response is made on the right-hand side than when it is made on the left-hand side, i.e., large size is associated with right and small size with left (Dehaene et al., 1993, p. 380). The brain seems to represent magnitude information spatially, like numbers on a ruler. On the basis of the results of Dehaene et al. (1993) and the notion of a metaphorical mental ruler, we believe that the conceptual distance between ingroup and outgroup may also be represented spatially, with the self located at the origin, on the left, with the ingroup nearby, and also on the left. We therefore argue that social categories may be spatially organized along a ‘social mental ruler’ representing the distance from the ingroup (on the left) to the outgroup (to the right). In particular, we hypothesized that when group membership is salient, people will consider ingroup as nearer to the self than outgroup. In line with this, we predicted that people when responding with the left hand (the hand nearer to the mental representation of self, i.e., the reference point), people would respond faster to an ingroup symbol than to an outgroup symbol and vice versa.

Before describing the three experiments carried out for this study, we offer a final comment on the distinction between the spatial organization of social categories (SOSC) and SAB. The SOSC, since it involves asymmetries in the LR mental representation of the social category presented on the center of the screen, predicts that left-hand responses should be faster to ingroup stimuli than to outgroup stimuli. Thus SOSC implies a mental map. The SAB predicts that responses to ingroup (more agentic) stimuli will be faster when they are presented on the left than when they are presented on the right (Maass and Russo, 2003, p. 299). In other words, SAB is based on explicit (physical) mapping of (agentic) stimuli. SOSC is also more general than SAB, as it is not limited to agentic aspects of social categories and should extend to all salient social categories. In addition SOSC is independent from the language schema (sequence of subject-object in active phrasing) and may be driven by simple images linked to the ingroup–outgroup categorization.

We tested our prediction that social categories are spatially organized in three experiments based on a left–right RT task. In Study 1 participants were assigned to teams on the basis of their favorite color and then asked to categorize faces according to whether they belonged to the same color team (ingroup) or the other team, using their hand and working as fast as possible. In Study 2 we attempted to replicate and generalize the findings of Study 1 using a different social category (nationality). Finally, in Study 3, we investigated the effects of conflict between the implicit spatial organization of social categories and their explicit positioning in physical space.

## Study 1^1^

### Materials and Methods

#### Participants

We calculated the required sample size as 18, based on the fact that the experimental design involved two repeated factors (response key: left vs. right and membership: ingroup vs. outgroup), the assumption (based on the results of Dehaene et al., 1993) that the interaction effect we hoped to observe would be modest ($\eta_{p}^{2}$ = 0.10, *f*= 0.33), a critical α of 0.05, a power (1-β) of 0.90, an assumed average correlation of 0.5 between the repeated measures and homogeneity of variances (ε = 1). A more pessimistic assumption about effect size ($\eta_{p}^{2}$= 0.05, *f* = 0.23) suggested a required sample size of 35 participants. A moderate violation of the assumption of homogeneity of variances (ε = 0.7) and a very pessimistic assumption about effect size ($\eta_{p}^{2}$ = 0.05, *f* = 0.23) would require a sample of 36 participants. We therefore recruited a sample of 45 English-speaking students (*F* = 16, *M* = 29, *M* age 23.53 years, *SD* = 3.38) via Prolific. All participants were right-handed and naive to the purpose of the experiment. Only mean correct response times were analyzed, so all participants with more than 15% incorrect responses were excluded from the analysis. Furthermore, all RTs lower than 250 ms and greater than 1000 ms (6.6% of all trials) were excluded from the analysis. Thus the final sample comprised 40 students (women: *n*= 13, *M* age = 23.23 years, *SD* = 3.17; men: *n* = 27, *M* age = 23.63 years, *SD* = 3.41). This study was carried out in accordance with the recommendations of Ethical Committee for Psychological Research at the University of Sapienza and all participants provided written, informed consent in accordance with the Declaration of Helsinki. The protocol was approved by the Ethical Committee for Psychological Research at the University of Sapienza.

#### Procedure and Materials

The whole procedure was carried out online. Participants were given detailed instructions about how to perform the experimental task. It was stressed that to perform well they would need to be in a quiet room with no distractions and should use computer with a QWERTY keyboard. This last requirement was necessary because the task required participants to use both hands to categorize social stimuli: the left-hand to press the ‘A’ key and the right-hand to press the ‘L’ key. Participants were told that their task was to categorize faces into two types - members of their own team and members of the other teams -as fast as possible. Group identity was induced in accordance with the minimal group paradigm (Rabbie and Horwitz, 1969; Tajfel et al., 1971). Thus before the categorization task participants were assigned to a color team on the basis of their color preference (they were asked to choose between five different colors: red, blue, green, orange, and black). At this point color team membership was made salient by informing participants that “*Participants that also [preferred the same color] showed to be really faster and more precise in the RT Task than participants assigned to [the other color] TEAM. Before assigning you to the [preferred] color-TEAM you have to show that you are also as fast and precise as members of the [preferred] color-TEAM are. So we invite you to perform the following RT Task and on the base of your performance we will decide whether we can make you part of [preferred] color-TEAM or not.*” After they had read this information participants were temporarily assigned to the team of their preferred color and another random color was chosen as the other team. Participants were then instructed to perform the training RT. At the end of this preliminary task a message informed them that their performance was in line with that expected for their preferred color team and they were assigned to that team.

**FIGURE 1:**
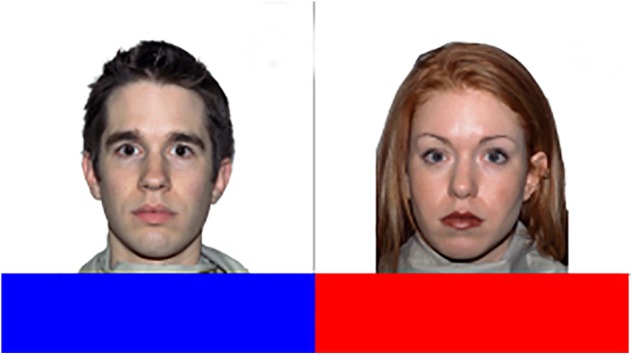
Example of Stimuli (Male, Female with two of the five possible color TEAM) used in the Social Categorization Task of Study 1 (face stimuli are taken from MacBrain Stimulus Set, Tottenham et al., 2009).

Next, participants were introduced to the Social Categorization Task. The stimuli (4.7° × 4.7°) were female or male faces with the color-TEAM positioned below the photograph (**Figure 1**) and were presented one at a time. Each trial started with the presentation of a blank screen for 1.5 s, then a fixation cross (0.5° × 0.5°) was presented in the center of the screen for 300 ms, followed by the target image. After a response (correct; incorrect) the next trial was presented. Each photograph was presented twice and the order of presentation was randomized. Images of six students of the same sex and ethnicity as the participant were assigned to the participant’s team and images of six other students were assigned to the other team (**Figure 1**). Thus a block of trials consisted of 24 trials and the task consisted of two experimental blocks (total of 48 trials). In one of the two blocks the images were presented in the center of the screen and participants were instructed to press the ‘A’ key with their left hand when a member of their own team appeared and to press the ‘L’ key with their right-hand when a member of the other team appeared; in the other block the response mapping was reversed and participants were asked to press the ‘A’ with the left when a member of the other color team appeared on the on the screen and to press the ‘L’ key with the right hand when a member of the preferred color team was presented. The trials in the block in which the participant’s team was associated with the left response key are referred to as ‘corresponding trials’ because we expected a match between the implicit spatial mapping of social categories and the response mapping of the task; those in the other block are referred to as ‘non-corresponding trials’ because we expected a conflict between the implicit spatial mapping of social categories and the response mapping of the task. The order in which the blocks were presented was counterbalanced across participants. The faces used as stimuli were selected from the MacBrain Face Stimulus Set^2^ (Tottenham et al., 2009), which consists of 646 facial expressions displayed by models varying with respect to gender and race for use in studies on emotion recognition. Each model in the Stimulus Set is shown displaying the following emotional expression: fearful, happy, sad, angry, surprised, calm, neutral, and disgusted. To control for potential confounding effects of gender, participants classified images of people of their own gender. We therefore selected two sets of stimuli: male and female. Each set consisted of 12 different Caucasian models displaying a neutral expression, 6 were assigned to the ingroup and 6 were assigned the outgroup. Stimuli were presented using the software package JATOS (Kühn and Filevich, 2015) and jsPsych libraries developed by de Leeuw (2015).

#### Measures

##### Assessment of ingroup favoritism

After being assigned to their preferred color team, participants were asked to describe how they felt about being part of it by rating six adjectives (happy; excited; motivated; disappointed; enthusiastic; full of energy) on a scale ranging from 1 (‘does not describe me at all’) to 5 (‘describes me completely’). Internal consistency for the measure was of 0.90 in this study. Participants also described their feelings about the other team using the same six items and the same rating scale. Reliability of the measure was 0.92. The minimal group paradigm procedure was successful in inducing the ingroup favoritism as the difference between ratings of the teams was statistically significant [*t*(39) = 4.85, *p* < 0.01, Cohen’s *d* = 0.58] with the preferred color team scoring higher (*M* = 3.04, *SD* = 0.77) than the other team (*M* = 2.56, *SD* = 0.87).

##### Reaction times

Only mean correct RTs between 250 and 1000 ms were considered. Participants with less than 85% correct responses were excluded.

### Results

**FIGURE 2:**
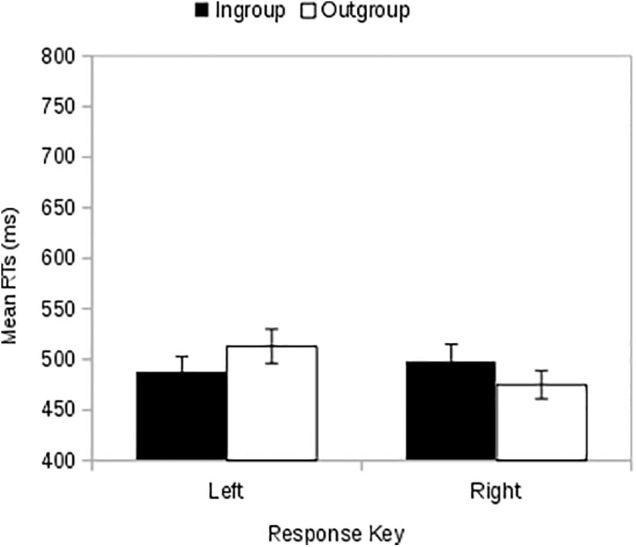
Study 1: When the ingroup stimuli is presented on the screen, participants are faster in responding with the left hand with respect to the right hand. Besides, when the outgroup stimuli are presented, right-hand responses are faster than the left-hand responses. Error bars indicate ±1 MSE.

Two-way ANOVA with response key (left vs. right) and membership (preferred color-TEAM = ingroup vs. other-color TEAM = outgroup) as within-subject factors revealed a significant interaction effect [*F*(1,39) = 7.79, *p* = 0.008, $\eta_{p}^{2}$ = 0.167; **Figure 2**). A simple effects analysis showed that the difference between ingroup and outgroup was significant both when we consider the left-hand responses [*F*(1,39) = 7.11, *p*= 0.011, $\eta_{p}^{2}$ = 0.154] with faster mean RTs when the left-key response was associated with the ingroup (*M* = 487, *MSE* = 16) than when it was associated with the outgroup (*M* = 513, *MSE* = 18), and when we consider the right-hand responses [*F*(1,39) = 4.37, *p* = 0.043, $\eta_{p}^{2}$ = 0.101] with faster RTs when the right key was associated with the outgroup (*M* = 475, *MSE* = 14) than when it was associated with the ingroup (*M* = 497, *MSE* = 17). There was no main effect of response key [*F*(1,39) = 3.84, *p =* 0.057, $\eta_{p}^{2}$ = 0.090] or membership [*F*(1,39) = 0.19, *p* = 0.667, $\eta_{p}^{2}$ = 0.005]. When a block sequence factor (block1 = ingroup-left response key; block2 = outgroup-left response key vs. block1 = outgroup-left response key; block2 = ingroup-left response key) was added to the ANOVA, the interaction between response key and membership remained significant [*F* (1,38) = 7.66, *p* = 0.009, $\eta_{p}^{2}$ = 0.168], and the three-way interaction (Response-Key by the Membership by the Block Sequence) was not significant [*F*(1,38) = 2.85, *p* = 0.100, $\eta_{p}^{2}$ = 0.070] and no interaction between block sequence and membership [*F*(1,38) = 0.13, *p* = 0.723, $\eta_{p}^{2}$ = 0.003] or response key [*F*(1,38) = 0.90, *p* = 0.348, $\eta_{p}^{2}$ = 0.023) was significant. Finally there was no main effect of block sequence [*F*(1,38) = 1.02, *p* = 0.319, $\eta_{p}^{2}$ = 0.026].

## Study 2

**FIGURE 3:**
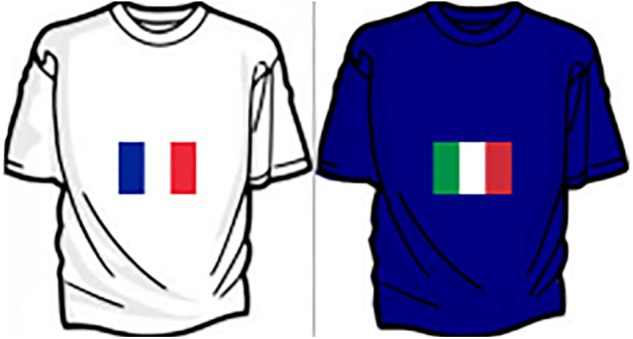
Example of Stimuli (National Flags: France vs. Italy) of the Social Categorization Task used in Study 2 and 3.

In Study 2 our aim was to demonstrate the generalization of the SOSC effect to a different social group. To do this, we treated nationality as a social category. The stimuli were images of t-shirts in different colors showing an image of the Italian or French flag (**Figure 3**).

### Materials and Methods

#### Participants

Forty-three Italian university students (*F* = 27; *M* = 16; *M* age = 20.77 years, *SD* = 0.89) who had not participated in the previous study were enrolled in this experiment. All participants were right-handed. Eight participants were subsequently excluded from analyses because their error rate was too high (greater than 15%). This study was carried out in accordance with the recommendations of Ethical Committee for Psychological Research at the University of Sapienza and all participants provided written, informed consent in accordance with the Declaration of Helsinki. The protocol was approved by the Ethical Committee for Psychological Research at the University of Sapienza.

#### Procedure and Materials

The experimental design was broadly the same as in Study 1, but the experiment was conducted in a laboratory rather than online and the stimuli were t-shirts depicting national flags (**Figure 3**) instead of faces. In addition participants were asked to complete a recall task by reporting how important it was for them to be identified as Italian rather than European, which was designed to make membership of the target social category (Italian nationality) salient. After the priming procedure all participants completed a nine-item social identification scale designed to measure the relative strength of individual and group identity (Hogg et al., 1998). Next, participants were introduced to the social categorization task. Stimulus presentation and data collection was controlled by an IBM Thinkcentre computer connected to a 17-inch monitor. Participants’ viewing distance was approximately 60 cm. Each trial started with a 1.5-s presentation of a blank screen followed by a 300-ms presentation of a central fixation cross (0.5° × 0.5°), after which the target image was presented. The next trial started once a response had been made. In this study the ingroup stimuli were images (4.7° × 4.7°, i.e., approximately the same size of stimuli as in Study 1) of t-shirts of six different colors depicting the Italian flag. T-shirts in the same six colors but showing the French flag were used as outgroup stimuli. All stimuli were presented twice per block, in a randomized sequence, yielding a total of 24 trials (12 ingroup trials; 12 outgroup trials). As in Study 1, the experimental task comprised two blocks of 24 experimental trials, yielding a total of 48 trials. In one of the two blocks participants were asked to press the left response key when the Italian flag appeared and the right response key when the French flag appeared; in the other block this association was reversed and participants were asked to press the left-key when the French flag appears on the screen and the right-key when the Italian flag appears on the screen. As in Study 1, the sequence of two blocks was randomized. The two blocks were preceded by a 12-trial training block (6 ingroup trials; 6 outgroup trials) in which participants were given feedback on the correctness of their categorization responses. Stimuli were presented using the software package PsychoPy 1.82.01 (Peirce, 2007, 2009).

#### Measures

##### Priming for identification

Participants were asked to recall and describe an especially salient event or a situation when they had felt proud to be Italian and then they were asked to describe how they felt by rating five emotional adjectives (‘happy’; ‘excited’; ‘motivated’; ‘realized’; ‘enthusiastic’) using a scale ranging from 1 (does not describe me at all) to 5 (describes me completely). The internal consistency (Cronbach’s alpha) for the priming index was 0.86 and the average priming score was *M* = 3.44 (*SD* = 0.86).

*Individual vs. Group Identification* (Hogg et al., 1998, p. 1256): a nine-item scale focusing on the importance for the self of and liking for, identification with, and a sense of belonging to a group (on a 9-point scale ranging from 1 not very much to 9 very much) was adapted to the present study to measure how much they identified with their nationality. Internal consistency reliability index was 0.91 with an average score of *M* = 6.28 (*SD* = 1.59) in the present study.

##### Reaction times

Mean RTs for correct responses were analyzed. RTs lower than 250 ms or greater than 1000 ms (5.4% of all trials) were excluded from analysis and data from participants with less than 85% correct responses were also excluded.

### Results

**FIGURE 4:**
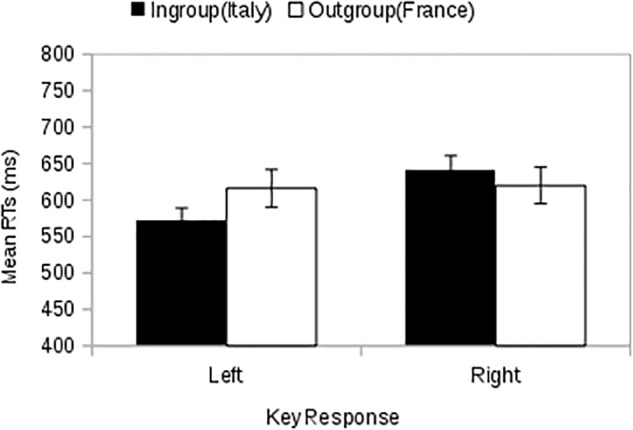
Study 2: The Spatial Organization of Social Categorization effect when the social categories are represented by Nationalities (Italy = Ingroup vs. France = Outgroup). Error bars indicate ±1 MSE.

Two-way ANOVA of RTs with membership and response key as within-subject factors revealed an SOSC effect qualified by the interaction between response key and membership [*F*(1,34) = 13.75, *p* < 0.001, $\eta_{p}^{2}$ = 0.288). A simple effects analysis showed that there was a difference between left-hand RTs to ingroup and outgroup stimuli (**Figure 4**) [*F*(1,34) = 22.08, *p* < 0.001, $\eta_{p}^{2}$ = 0.394], with faster RTs to ingroup images (*M =*571, *MSE =*18) than to outgroup images (*M =*616, *MSE =*21). However, there was no difference between right-hand RTs to ingroup and outgroup stimuli [*F*(1,34) = 2.82, *p* = 0.102, $\eta_{p}^{2}$ = 0.077] with faster RTs when outgroup images are presented (*M*= 591, *MSE*= 19) than when ingroup images are presented (*M*= 608, *MSE*= 19). There was a main effect of membership [*F*(1,34) = 5.78, *p* = 0.022, $\eta_{p}^{2}$ = 0.145], with faster RTs to ingroup stimuli (*M* = 590, *MSE* = 17) than to outgroup stimuli (*M* = 603, *MSE* = 19). Finally, there was no effect of response key [*F*(1,34) = 0.45, *p* = 0.507, $\eta_{p}^{2}$ = 0.013). As in Study 1 we repeated the analysis including a block sequence factor. There was no three-way membership by response key by block sequence interaction [*F*(1,33) = 1.21, *p* = 0.280, $\eta_{p}^{2}$ = 0.035) and the interaction between membership and response key remained significant [*F* (1,33) = 13.59, *p* = 0.001, $\eta_{p}^{2}$ = 0.292]. There was no interaction between block sequence and membership [*F*(1,33) = 1.18, *p* = 0.286, $\eta_{p}^{2}$ = 0.034) or response key [*F*(1,33) = 0.03, *p* = 0.870, $\eta_{p}^{2}$ = 0.001]; however, there was a main effect of block sequence [*F*(1,33) = 20.82, *p* < 0.001, $\eta_{p}^{2}$ = 0.387] such that participants responded more quickly when the left response key was associated with the outgroup in the first block (*M* = 530, *MSE* = 20) than when it was associated with the ingroup (*M* = 659, *MSE* = 20).

## Study 3

**FIGURE 5:**
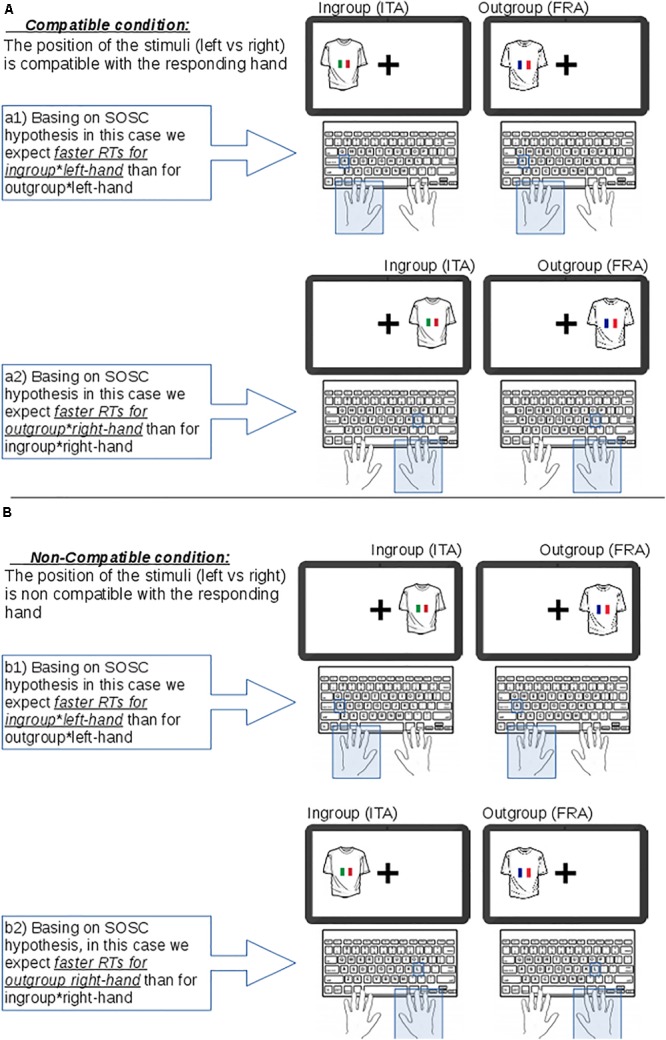
Description of the compatible and non-compatible conditions used in Experiment 3. **(A)** In compatible condition there is a match between the responding hand and the position of the social category on the screen. **(B)** In the non-compatible condition the image is presented on the opposite side of the responding hand.

In Studies 1 and 2 we collected empirical evidence that the SOSC effect applied to two different social categories. In Study 3 we used the same stimuli and task format as in Study 2, but we also manipulated the location of stimulus presentation, i.e., stimuli were no longer presented in the center of the screen but on the left or right side of the screen. By varying the position of stimuli in the visual field, we were able to investigate whether the implicit LR representation of social categories (respectively ingroup and outgroup) was modulated by the explicit position of social categories in physical space (i.e., on the left or right side of the visual field). In other words, in our first two experiments we examined whether the implicit mapping activated by the SOSC effect emerges when social categories are presented in the center of the screen. This mapping is implicit as it represents the mental arrangement of social categories along a hypothetical social continuum with the ingroup on the left and the outgroup on the right. Because the stimuli were presented in the center of the screen in the first two experiments, we do not know whether the SOSC effect can still be observed when this implicit mapping conflicts with the explicit physical (Left–Right) positioning of the stimuli. In particular we were interested in comparing conditions in which the stimulus was displayed on the same side as the correct response key (compatible response) or on the opposite side from the correct response key (incompatible response). These two conditions are illustrated in **Figure 5**, where the upper panel shows two pairs of compatible conditions in which ingroup or outgroup images are presented on the left of the screen and are associated with the left-hand response (**Figure 5**, a1 conditions) or presented on the right of the screen and associated with the right-hand response (**Figure 5**, a2 conditions). There are also two pairs of incompatible conditions: the first involves pairing an ingroup or outgroup images on the right of the screen with a left-hand response (**Figure 5**, b1 conditions) and the second involves pairing an ingroup or outgroup image on the left of the screen with the right-hand response (**Figure 5**, b2 conditions). If the SOSC effect applies then we would expect left-hand RTs to left-screen images of the ingroup to be faster than left-hand RTs to left-screen images of the outgroup and correspondingly right-hand RTs to right-screen outgroup images to be faster than right-hand RTs to right-screen ingroup images. It is not clear, however, what the pattern of responses would be in the two pairs of incompatible conditions. If the SOSC effect is independent of the explicit position of stimuli then we would expect that left-hand responses would be faster to ingroup stimuli than to outgroup stimuli, regardless of the position of the stimuli on the screen. In particular we would expect participants to be faster in responding with left-hand to ingroup images than to outgroup images presented on the right of the screen. Correspondingly we expect participants to be faster also when responding with the right-hand to outgroup images than to ingroup images presented on the left side of the screen. On the contrary if the SOSC effect is dependent on the explicit position of the stimuli then the association between responding hand and category membership should not apply in the incompatible conditions.

When it comes to the SNARC effect, Notebaert et al. (2006) have already investigated whether implicit and explicit spatial information share the same spatial representation and found that stimulus location modulated the SNARC effect: a typical SNARC effect was observed when the implicit spatial mapping rule was compatible with the explicit spatial information whereas a reversed SNARC effect was observed when the implicit spatial mapping rule was incompatible with the explicit spatial information. According to Notebaert et al. (2006) this result was due to the fact that both types of spatial information (implicit and explicit) activate the same spatial representation. A similar line of reasoning can be applied to our Experiment 3: as already stated, if an SOSC effect were observed in both compatible and incompatible conditions (**Figure 5**) we would conclude that the implicit representation of SOSC is independent from the explicit positions of stimuli. On the contrary if an SOSC effect were observed in the compatible conditions and a reversed SOSC effect in the incompatible conditions then we would conclude that that the implicit position of ingroup and outgroup stimuli activated by the SOSC effect shares the same spatial representation of the explicit position of the same stimuli.

Manipulating the positions of the stimuli and the responding hand also raised the possibility that we would observe a Simon effect (Simon, 1969). The Simon effect occurs when participants respond faster and more accurately when an irrelevant position stimulus (for example, left side of the screen) is matched with the response (for example, left key-press) than when it does not match with the response key. In our experiment, a Simon effect would manifest as faster responding when using the hand on the same side as the stimulus (i.e., responses to left-screen stimuli would be faster with the left hand than with the right and vice versa), independently of social category. In this case, the explicit position of the stimulus activates the nearer hand and explicit mapping of stimulus-hand takes precedence over implicit mapping in responding to the stimulus, independently of social category.

We also believe that interference between implicit and explicit mapping can be used to disentangle the SOSC effect from the SAB effect. The SAB effect involve more agentic categories being represented on the left of a mental continuum and is related to the structure of active phrases – the subject (the agent) precedes the object. In other words, the SAB is triggered by the explicit position of (agentic) stimuli. In our Study 3, the SAB effect should manifest as faster responding to ingroup stimuli than to outgroup stimuli when located on the left and should be independent of the laterality of the response. So if the SAB is active we would find a significant interaction between the social category and the side of presentation independently of the responding hand. On the contrary, the SOSC effect predicts that respondents should be faster not only when the ingroup category is associated with a left-hand response but also when the outgroup is associated with the right-hand response. In other words the SOSC effect depends on the responding hand, not the stimulus location. Hence, if there is an SAB effect then we would observe an interaction between social category and stimulus location.

Study 3 thus allowed us to test not only whether implicit mapping and explicit mapping share the same spatial representation but also will allow us to disentangle the SOSC effect from the SAB effect.

### Materials and Methods

#### Participants

The sample comprised 50 right-handed students (*F* = 29, *M* = 21; *M* age = 20.62 years, *SD* = 0.75) who had not taken part in any of the preceding studies. Four participants were excluded from the analysis because they responded incorrectly to an entire block of trials or had too high an error rate (>15%). The final sample thus comprised 46 participants. This study was carried out in accordance with the recommendations of the Ethical Committee for Psychological Research at the University of Sapienza and all participants provided written, informed consent in accordance with the Declaration of Helsinki. The protocol was approved by the Ethical Committee for Psychological Research at the University of Sapienza.

#### Procedure and Materials

Participants were presented with a series of stimuli associated with the ingroup or outgroup, as in the previous studies, but in this experiment they were presented to the left or right side of the screen, rather than in the center. Thus there were now three experimental factors: social category (ingroup vs. outgroup), responding hand (left vs. right) and presentation side (left vs. right). As described above this yielded eight conditions, four ‘compatible’ and four ‘incompatible.’ The compatible conditions (**Figure 5**, conditions a1 and a2) are those where the stimulus is presented on the same side as the correct response. So we have the images (whether ingroup or outgroup) presented on the left and the responding hand is left (**Figure 5**, a1); or images (whether ingroup or outgroup) presented on the right and the responding hand is the right (**Figure 5**, a2). The incompatible conditions are those conditions where the images (ingroup or outgroup) are presented on the opposite side to the correct response (**Figure 5**, b1: right-side stimulus, left response correct; b2: left-side stimulus, right response correct).

The experimental task was divided into two blocks of 24 trials and the stimuli appear to the left or on the right of the central fixation point. In one of the two blocks (spatially compatible condition) participants had to press the left response key when the Italian flag appeared on the screen and the right response key when the French flag appeared, regardless of the location of the flag. In the spatially incompatible block this association was reversed, so that participants had to press the right response key when the Italian flag was shown and the left response key when the French flag was shown, regardless of the location in which the flag appeared. Each stimulus type appeared the same number of times and each location and the order of locations was balanced and randomized. Block order was counterbalanced so half the participants performed the spatially compatible block first and half performed the spatially incompatible block first. The experimental blocks were preceded by a training block in which participants were given feedback about their responses. The upper and lower RT limits were the same as in Studies 1 and 2 (250 ms; 1000 ms) and RTs fell outside these limits on about 6.5% of all trials. The procedure and measures were the same as in the Study 2, but the experimental design included stimulus position (left; right) as an additional experimental factor. The stimuli were images of t-shirts showing the French or Italian national flag, as in Study 2, but were presented to the left or right side of the screen. Also as in Study 2 the experimental task was preceded by a recall task designed to make the target social category salient. We used the same priming procedure as in Study 2.

### Results

**FIGURE 6:**
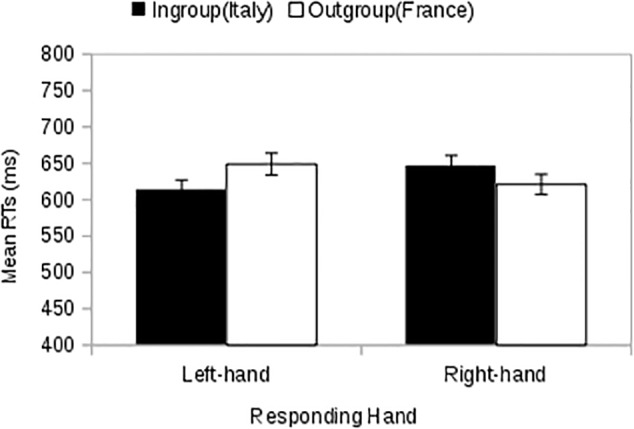
Study 3: The interaction between social category (Ingroup vs. Outgroup) with the responding hand (left-hand vs. right-hand) independently of whether the side of presentation of stimuli matches the responding hand (compatible conditions) or not (non-compatible conditions). Error bars indicate ±1 MSE.

**FIGURE 7:**
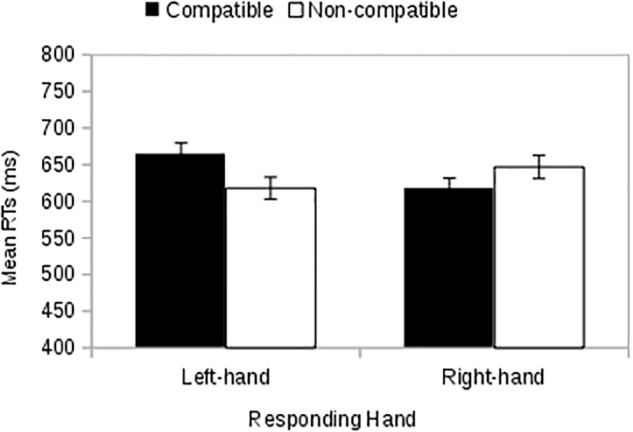
Study 3: The interaction between responding hand (left hand vs. right hand) and the compatibility factor (compatible vs. non-compatible), independent of whether the social category is ingroup or outgroup. Error bars indicate ±1 MSE.

The full factorial design in experiment 3 is a Social Category (ingroup vs. outgroup) × Hand (left vs. right) × Compatibility (compatible vs. non-compatible) factorial design. The three-way interaction was not significant [*F*(1,45) = 1.61, *p* = 0.211, $\eta_{p}^{2}$ = 0.034]; other results, i.e., simple effects analysis of the three-way interaction are discussed in the Supplementary Material. There was a two-way interaction between social category (ingroup vs. outgroup) with the responding hand (left-hand vs. right-hand) [*F*(1,45) = 20.24, *p* < 0.001, $\eta_{p}^{2}$ = 0.308] confirming the presence of an SOSC effect that was independent of whether the stimulus appeared on the same side as the correct response key. In particular (**Figure 6**) we found that participants’ left-hand responses were faster [*F*(1,45) = 18.89, *p* < 0.001, $\eta_{p}^{2}$ = 0.296] when they were responding to an ingroup stimulus (*M* = 613, *MSE* = 14) than when they were responding to an outgroup stimulus (*M* = 646, *MSE* = 15), whilst the opposite pattern applied to right-hand responding: participants were faster [*F*(1,45) = 11.58, *p* = 0.001, $\eta_{p}^{2}$ = 0.205] in responding to outgroup stimuli (*M* = 621, *MSE* = 14) than ingroup stimuli (*M* = 649, *MSE* = 15). There was also an interaction between responding hand and compatibility [*F*(1,45) = 4.12, *p* = 0.048, $\eta_{p}^{2}$ = 0.084). A simple effects analysis showed (**Figure 7**) that when responding with their left hand, participants were faster [*F*(1,45) = 5.25, *p* = 0.027, $\eta_{p}^{2}$ = 0.104] in compatible conditions (*M* = 621, *MSE* = 14) than in incompatible conditions (*M* = 641, *MSE* = 15), but when responding with their right hand, RTs were similar [*F*(1,45) = 0.21, *p* = 0.649, $\eta_{p}^{2}$ = 0.005) in compatible conditions (*M* = 635, *MSE* = 15) and incompatible conditions (*M* = 632, *MSE* = 14). There was no interaction between social category and compatibility factor [*F*(1,45) = 0.23, *p* = 0.632, $\eta_{p}^{2}$ = 0.005] and no main effect of social category [*F*(1,45) = 2.23, *p* = 0.142, $\eta_{p}^{2}$ = 0.047]; responding hand [*F*(1,45) = 0.39, *p* = 0.536, $\eta_{p}^{2}$ = 0.009]; or compatibility [*F*(1,45) = 2.49, *p* = 0.122, $\eta_{p}^{2}$ = 0.052].

Our second hypothesis related to whether the SAB effect was activated in the social category task with variable stimulus position. Compatibility was irrelevant to this hypothesis so we ran a three-way ANOVA with social category, responding hand, and stimulus location to determine whether the SAB effect emerged. There was no interaction between social category and stimulus location [*F*(1,45) = 1.61, *p* = 0.211, $\eta_{p}^{2}$ = 0.034) so we can exclude the possibility of an SAB effect. Also the Simon effect, i.e., the interaction between responding hand and stimulus location, was not significant [*F*(1,45) = 2.49, *p* = 0.122, $\eta_{p}^{2}$ = 0.052]. A more thorough presentation and discussion of these results may be found in the Supplementary Material.

### Discussion

There is already evidence that ingroups and outgroups are processed differently and that the self represents the “habitual reference of point” (Srull and Gaelick, 1983, p. 111; Aron et al., 1991; Smith et al., 1999). Moreover, studies of spatial bias in various areas of psychology have consistently shown that in Western cultures people tend to order quantity from left to right (smaller quantities are represented on the left; Dehaene et al., 1993; Gevers et al., 2004). The preference for positioning the sentence subject to the left (Chatterjee, 2002) has led some authors to advance the hypothesis that people also tend to envisage intergroup situations (ingroup/outgroup) following a LR ordering, an effect also known as spatial agency bias (Maass and Russo, 2003; Maass et al., 2009). Drawing on these studies on spatial biases and inspired by the literature on the SNARC effect (Dehaene et al., 1993), we hypothesized that there is also a spatial dimension to mental representation of social categories, with ingroups represented on the left of a social continuum and outgroups on the right.

Evidence from Studies 1 and 2 led us to conclude that ingroups and outgroups are processed differently when the group membership is salient. In particular, the overall pattern of RTs in both experiments show a spatial effect such that when responding with the left hand, responses to ingroup stimuli were faster than responses to outgroup stimuli and vice versa. It should be noted, however, that whilst responses in Study 1 conformed exactly to this pattern, this was not the case in Study 2. In Study 2, left-hand responses were faster to ingroup stimuli than outgroup stimuli, but right-hand RTs were not affected by social category. One possible explanation for the difference between the pattern of responding in Studies 1 and 2 is the difference in the procedure used to make social category salient. In Study 1 participants were assigned to a team on the basis of their color preference and then asked to perform an RT task to prove that they had the requisite characteristics for membership of that team, whereas in Study 2 participants were asked to recall a memorable past experience when they had felt to be proud to be Italian. The first paradigm appears to be more effective than the second, presumably because it is not based on personal experiences, which may vary dramatically from person to person.

Just as Dehaene et al. (1993) interpreted their results in terms of a spatial bias in representation of quantities (SNARC effect), we interpreted the differential processing of social categories in terms of spatial bias. We posit that the social category that is salient at a specific moment is automatically positioned on the left of the mental map whilst the other categories are positioned on the right. The spatial arrangement of social categories is dynamic rather than fixed and determined on the basis of salience as we can see when we compare the results of Studies 1 and 2. Different paradigms were used to activate group membership: in Study 1 we used the classical minimal group procedure to induce intergroup differences on the basis of color preference, whereas in Study 2 we used rehearsal of personal experience to activate nationality as a social category. Our results are consistent with Schubert and Otten’s (2002) suggestion that when a social category becomes salient this alters the ratio between intragroup differences and intergroup differences and consequently ingroups and outgroups are processed differently. Intragroup difference is the degree of difference between self-identity and group identity, whereas intergroup difference is the degree of difference between ingroup and outgroup at the superordinate level (Schubert and Otten, 2002). When the membership of a particular social category becomes salient, people tend to associate themselves more strongly with that social category (i.e., intragroup difference decreases) and to differentiate the relevant ingroup more strongly from outgroups (i.e., intergroup differences decrease with respect to the salient category). Our results show that this social bias is reflected in the implicit spatial representation of categories: the ingroup (and hence the self) is represented on the left and the outgroup on the far right. The SOSC thus seems to be consistent with the main predictions of meta-contrast principle (Turner et al., 1987; Oakes et al., 1999).

The results relating to spatial ordering of social categories cannot be explained in terms of familiarity with the stimuli. Participants did not know any of the people whose faces were used as ingroup and outgroup stimuli, so the spatial difference in RTs to ingroup faces cannot be explained in terms of familiarity with those faces. This strengthens our speculation that participants processed ingroup and outgroup faces differently.

Handedness can also be excluded as a possible explanation of the LR asymmetry we found, as in the case of SNARC. All participants were right-handed and if a handedness bias had been present we would have expected to observe a main effect of the responding hand, with faster RTs when using the right hand, independently of the social category of the stimulus. No such main effect was observed in any of the experiments, and so we conclude that the SOSC effect we observed was not due to handedness.

Study 3 demonstrated that the SOSC effect was independent of the physical position of the social category stimuli. Thus, in contrast to the findings of Notebaert et al. (2006) in relation to the SNARC effect, the results of Study 3 imply that simple social categorization automatically prompts a representation of stimuli that it is spatially ordered and that is not affected by the position of stimuli in physical space. So if only one implicit mapping is allowed, one would predict that the SOSC effect would disappear when two alternative ingroup categories are both salient and competing for the same representational mapping. In this case, participants would have to deal with two conflicting implicit mappings where two possible ingroups compete for being located on the left. Furthermore, the results of all three experiments also help to differentiate SOSC from SAB in different ways. In Study 1 we used faces and team colors as stimuli and although we matched the gender of participants and stimulus faces in order to control for stereotypical effect often found in SAB studies (Maass et al., 2009), we cannot exclude the possibility that the SAB effect was automatically activated because ingroup faces were considered more agentic than outgroup faces. Moreover, in Study 2, which used different stimuli and different social categories, we did not find a main effect of social category or response key, which implies that any SAB effect plays only a marginal role in the SOSC effect. Finally in Study 3 we used national flags as stimuli in an experiment in which the implicit spatial coding of social category conflicted with the physical position of the stimuli in some trials and also failed to observe an SAB effect, confirming that ingroup national flags are not perceived as more agentic than outgroup national flags. Taken together the results of Studies 1 and 2 suggest that the SOSC effect is a social spatial bias with a different basis from the SAB effect.

Future research should be aimed at exploring which factors affect the spatial ordering of social categories. A first possibility is that some social groups (e.g., family, close friends) may be more easily associated with the self than with others (based on, for e.g., nationality, attendance at the same university). Do these social groups (e.g., family) automatically prompt spatial ordering or is salience still critical? Group size may affect the SOSC effect; it is one of the components of the ‘entitativity’ construct (Sherman et al., 1999; Lickel et al., 2000; Castano et al., 2003) which, along with others (for a review see Lickel et al., 2000) represents the degree to which social groups are perceived as real units (Castano et al., 2003) and as structured and important groups (Sherman et al., 1999). Even if evidences supporting the relationship between the dimension of group and entitativity are not conclusive (Lickel et al., 2000), most social psychologists agree that the larger a group, the less it will be perceived as entitative (see also Lickel et al., 2001). Given that people tend to identify more strongly with small groups and highly entitative groups (Castano et al., 2002, 2003; Johnson et al., 2006; Crawford and Salaman, 2012), such as family or very close friends, we suggest that the size of groups may also potentially affect the spatial organization of social groups. We also speculate that, as with quantity, small and highly entitative groups tend to be positioned to the left of larger, less entitative groups.

Although further replications and evidence of generalization to other social categories are needed, these findings have important practical implications. In particular, the social categorization task may be useful as a method of determining which social categorization takes precedence and may have a role to play in the measurement of ingroup bias. A more interesting application is that the SOSC effect introduces the possibility of reducing the negative effect of intergroup differentiation through (spatial association) training on counter-categorization as showed in a recent work by Hu et al. (2015) that proved effective for the memory consolidation of training effects (Buzsák, 1998; Stickgold, 2005; Backhaus et al., 2008).

## Author Contributions

Conceived and designed the experiments: FP and MR. Performed the experiments: FP and MR. Analyzed the data: FP and MR. Wrote the first draft of the paper: FP and MR.

## Conflict of Interest Statement

The authors declare that the research was conducted in the absence of any commercial or financial relationships that could be construed as a potential conflict of interest.

## Footnotes

1 Data of the three experiments are available at the following web address: https://osf.io/w3udk/

2 http://www.macbrain.org/resources.htm
