# Supplementary material for: Is Social Categorization Spatially Organized in a “Mental Line”? Empirical Evidences for Spatial Bias in Intergroup Differentiation
Source: Front Psychol. 2018 Feb 15;9:152. doi: 10.3389/fpsyg.2018.00152 (PMC5818433; doi:10.3389/fpsyg.2018.00152)
Supplement: Supplementary file 1 [file Data_Sheet_1.doc]

SUPPLEMENTARY MATERIAL TO EXPERIMENT 3

In Experiment 3 we found that the triple interaction effect between Social Category (Ingroup vs. Outgroup) x Hand (Left vs. Right) x Compatibility (Compatible vs. Non-compatible) was not significant (*F*(1, 45) = 1.61, *p* = 0.211, *η^2^_P_* = 0.034). However even if the interaction effect is not significant this does not mean that the simple interaction effects representing the SOSC effect (i.e. the Social Category X Hand interaction) is indeed present in both the compatible condition as well as the non-compatible condition. So, in the following we present results of this post-hoc analysis. By decomposing the triple interaction effect we found that in the compatible condition (figure 1s), the left-hand was faster (*F*(1, 45) = 4.49, *p* = 0.040, *η^2^_P_* = 0.091) in reacting to ingroup (*M* = 607, *MSE* = 15) than to outgroup (*M* = 635, *MSE* = 16), while the right hand reported faster RTs (*F*(1, 45) = 4.86, *p* = 0.034, *η^2^_P_* = 0.096) to outgroup stimuli (*M* = 625, *MSE* = 15) than to ingroup stimuli (*M* = 645, *MSE* = 16). In the non-compatible condition (figure 2s) we found a similar pattern of results and in particular we found that left-hand RTs were faster (*F*(1, 45) = 21.72, *p* < 0.001, *η^2^_P_* = 0.326) when the ingroup stimuli were presented on the screen (*M* = 618, *MSE* = 15) than when outgroup stimuli are presented (M = 664, MSE = 16) and finally right hand RTs were faster (*F*(1, 45) = 9.98, *p* = 0.003, *η^2^_P_* = 0.182) with outgroup stimuli (*M* = 617, *MSE* = 15) than with ingroup stimuli (*M* = 664, *MSE* = 16).

Moreover to further show that the spatial position of stimuli (explicit map) does not affect the implicit map we decomposed the triple interaction effect by considering the interaction between Social Category and Compatibility when participants respond with the left-hand and when they respond with the right-hand. When we consider the Left-hand average RTs to ingroup stimuli we found no significant difference (*F*(1, 45) = 0.91, *p* = 0.345, *η^2^_P_* = 0.020) between compatible (M = 607, MSE = 15) and non-compatible conditions (M = 618, MSE = 15). However when we consider Left-hand average RTs to outgroup stimuli we found a significant difference (*F*(1, 45) = 5.69, *p* = 0.021, *η^2^_P_* = 0.112) between compatible (M = 635, MSE = 16) and non-compatible conditions (M = 664, MSE = 16). Considering Right-hand average RTs to ingroup stimuli again we found no significant differences (*F*(1, 45) = 0.06, *p* = 0.800, *η^2^_P_* = 0.001) between compatible (M = 645, MSE = 16) and non-compatible conditions (M = 647, MSE = 16) and a similar patter was also found when considering Right-hand average RTs to outgroup stimuli with no significant differences (*F*(1, 45) = 0.64, *p* = 0.426, *η^2^_P_* = 0.014) between compatible (M = 625, MSE = 15) and non-compatible conditions (M = 617, MSE = 15). Given that in just one case the effect was significant, this pattern of results mainly support the conclusion that the spatial position of stimuli does not affect the implicit map derived from the SOSC.

**Results concerning the Social Category by Hand by Position ANOVA**

For the present experiment we manipulated three factors, Group x Hand x Position, for investigating whether the position of stimuli may affect the SOSC effect. Similar to Notebaert et al (2006), who explored this question for the SNARC effect, we rearranged the conditions so as to obtain a compatibility factor (figure 5). This re-arrangement allowed us to investigate whether we observe the SOSC (group x hand interaction) when the position of stimuli is compatible with (i.e. the same of) the responding hand (compatible condition) or when the position of the stimuli is not compatible with the responding hand (non-compatible condition). Standing this premises, however the Group x Hand x Position design (Please refer to Figure 5 for envisaging how the ANOVA design would change using the Position factor on the behalf of the Compatibility factor) may be useful for testing a different set of hypotheses concerning the two-way interaction effects. In such ANOVA design the two way interaction effects investigate the following hypothesis: the Group x Hand interaction tests the SOSC effect; the Group x Side interaction tests the SAB effect; and finally the Hand x Side interaction effect tests the Simon effect. The triple interaction (Group x Hand x Position) effect was not significant (*F*(1, 45) = 0.23, *p* = 0.632, *η^2^_P_* = 0.005) as well as the two-way interaction effects of Group x Side (i.e. the SAB effect; *F*(1, 45) = 1.61, *p* = 0.211, *η^2^_P_* = 0.034) and of the Hand x Side (i.e. the Simon effect; *F*(1, 45) = 2.49, *p* = 0.122, *η^2^_P_* = 0.052). However the interaction effect of Group x Hand (i.e. the SOSC effect) is significant (*F*(1, 45) = 20.02, *p* < 0.001, *η^2^_P_* = 0.308). Finally also the main effects of Group (*F*(1, 45) = 2.23, *p* = 0.142, *η^2^_P_* = 0.047) and of Hand (*F*(1, 45) = 0.39, *p* = 0.536, *η^2^_P_* = 0.009) were not significant. However the main effect of Side was significant (*F*(1, 45) = 4.12, *p* = 0.048, *η^2^_P_* = 0.084) with the faster average RTs when the stimuli are presented on the Left side of the screen (*M* = 627, *MSE* = 14) than when stimuli are presented on the Right side of the screen (*M* = 638, *MSE* = 14).

Figure 1s – Means and MSE for the Social Category (ingroup vs outgroup) by Hand (Left vs Right) interaction effect in the Compatible Condition

Figure 2s – Means and MSE for the Social Category (ingroup vs outgroup) by Hand (Left vs Right) interaction effect in the Non-Compatible Condition
