# Supplementary figures and images for: Is Social Categorization Spatially Organized in a “Mental Line”? Empirical Evidences for Spatial Bias in Intergroup Differentiation
Source: Front Psychol. 2018 Feb 15;9:152. doi: 10.3389/fpsyg.2018.00152 (PMC5818433; doi:10.3389/fpsyg.2018.00152)

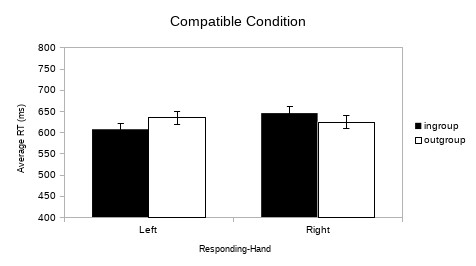

Supplement: Supplementary file 2 [file Image_1.TIF]

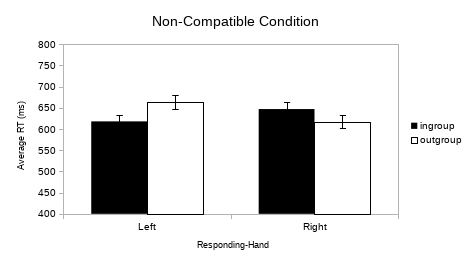

Supplement: Supplementary file 3 [file Image_2.TIF]
